# Kawasaki disease: Clinico-laboratory spectrum and outcome in a cohort of children treated at a tertiary care hospital in Islamabad, Pakistan

**DOI:** 10.12669/pjms.36.2.910

**Published:** 2020

**Authors:** Seema Sakina, Syeda Sobya Owais, Ejaz Ahmed Khan, Abdul Malik Sheikh

**Affiliations:** 1 Seema Sakina, MBBS. Post Graduate Resident, Department of Pediatrics, Shifa International Hospital and Shifa Tameer-e-Millat University, H-8/4, Islamabad, Pakistan; 2 Syeda Sobya Owais, MD. Associate Consultant Pediatrician, Emergency Department, Shifa International Hospital and Shifa Tameer-e-Millat University, H-8/4, Islamabad, Pakistan; 3 Ejaz Ahmed Khan, MD. Chief of Pediatrics, Shifa International Hospital and Shifa Tameer-e-Millat University, H-8/4, Islamabad, Pakistan; 4 Abdul Malik Sheikh, FCPS. Associate Consultant Pediatric Cardiologist. Shifa International Hospital and Shifa Tameer-e-Millat University, H-8/4, Islamabad, Pakistan

**Keywords:** Children, Coronary artery disease, Kawasaki disease

## Abstract

**Objective:**

To describe the demographics; clinical, laboratory, echocardiographic findings; treatment and outcome in a cohort of children with Kawasaki disease in a tertiary care hospital.

**Methods:**

This is a descriptive, observational, retrospective cohort study conducted at Shifa International Hospital, Islamabad, from January 2013-June 2019. Children who met the criteria for Kawasaki disease according to the American Heart Association and American Academy of Pediatrics guidelines were included.

**Results:**

A total of 25 children who met the criteria of Kawasaki disease were included. Their mean age was 43 months (4-150 months). Majority (76%) were males. Eighteen (72%) had complete Kawasaki disease and 7 (28%) had incomplete Kawasaki disease. Fever (> five days) was present in 20 (80%) patients. Eight patients (32%) had echocardiographic changes, out of which two patients (25%) had complete Kawasaki disease and six patients (75%) had incomplete Kawasaki disease. Intravenous immunoglobulin was given to all patients. Fifteen children (60%) received intravenous immunoglobulin within 10 days of fever. None required a second dose. All patients received high dose aspirin at diagnosis which was reduced to antiplatelet dose after resolution of fever for ≥48hrs. Eighteen patients (72%) came for regular follow up. Follow up at 6 months showed complete resolution of echocardiographic changes in six patients (75%), 1 (12.5%) was lost to follow up and one (12.5%) child had persistent coronary artery dilatation.

**Conclusion:**

Complete KD was present in 72% of our cohort of children. Coronary artery abnormalities were present in one third of these children, at younger age and more common in those with incomplete KD but recovered in most.

## INTRODUCTION

Kawasaki disease (KD) is an acute, self-limiting disease of unknown etiology.^1^,^2^ It involves both small and medium-sized vessels of various organs with predilection for the coronary arteries.^2^,^3^ It is mainly a childhood illness, predominantly in <5 years old, occurring in all races around the world.^4^

The diagnosis of KD is made on criteria based on clinical signs and symptoms.^5^ KD can lead to coronary artery aneurysms in 25% of untreated cases.^6^ Treatment with intravenous immunoglobulin (IVIG) and aspirin reduces the risk of coronary artery abnormalities (CAA) when administered within 10 days of fever onset.^1^,^5^,^6^ Early recognition and management of KD is crucial in preventing long-term cardiac sequelae.

KD is the leading cause of childhood acquired heart disease in developed countries.^6^ Although KD is known to occur in India,^3^,^7^ studies from Pakistan are scarce. Only one study from Pakistan looked at the pattern of cardiovascular involvement in Pakistani children with KD.^8^

This study is meant to share the common epidemiological and clinical features, laboratory and echocardiographic parameters, as well as treatment and outcome of KD in children presenting to our center. The aim is to promote its awareness and help in prevention of its complications.

## METHODS

This is a descriptive, observational and retrospective cohort study. The electronic medical records database of Shifa International Hospital was searched for children with the diagnosis of KD from pediatric and cardiology clinics, inpatient and emergency department between January 2013 to June 2019. This study period was chosen as record keeping of patients with the diagnosis of KD was started in 2013 and collected up till date. Approval (Ref. IRB# 106-596-2019 dated April 29, 2019) was granted by the hospital Institutional Review Board (IRB) and Ethics Committee. All patients ≤ 16 years who met the criteria for KD according to the American Heart Association and American Academy of Pediatrics guidelines were included in the study. The clinical criteria for complete KD included fever ≥5 days and at least four of the five principal clinical features: polymorphous rash, non purulent conjunctivitis, cervical lymph node enlargement, changes of the extremities and changes in the oral mucosa). Incomplete KD was defined as, fever lasting for at least five days and at least two of the five clinical criteria for KD with absence of any other reasonable explanation for the illness and laboratory findings consistent with severe systemic inflammation.^5^

Clinical notes were reviewed with respect to patient demographics, clinical features, investigations, echocardiographic findings, treatment and follow-up. Investigations included complete blood count, and inflammatory markers erythrocyte sedimentation rate (ESR) and C-reactive protein (CRP).

Echocardiography was performed by a pediatric cardiologist using GE vivid 7 and Philips Sonos 5500 echocardiographic machines. Those with coronary artery involvement on initial echocardiogram remained on long-term follow-up for at least 12 months with clinical examination and echocardiogram at regular intervals every two to four weeks until resolution of the echocardiographic findings. As there is no data of coronary artery dimensions in Pakistani children, definition of coronary lesions was based on the criteria defined by The Research Committee of Kawasaki disease sponsored by the Ministry of Health and Welfare of the Japanese government.^9^ They defined coronary artery dilation as any coronary artery branch diameter measuring ≥3 mm in children <5 years old, or >4 mm in children ≥5 years old, or if the internal diameter of any branch was 1.5-fold greater than any adjacent segment. Patients with internal lumen diameter between 5 and 8 mm were classified as having non-giant aneurysms and those with lesions >8 mm were classified as having giant aneurysms.^9^

All patients were treated with intravenous immunoglobulin (IVIG), regardless of day of presentation, at a dose of 2gm/kg in divided doses, each administered over 6hrs, with monitoring of blood pressure**.** All children received high dose aspirin at a dose of 80-100 mg/kg at the time of diagnosis, which was reduced to low antiplatelet dose (3–5 mg/kg/day) when the patient was afebrile for ≥ 48 hours, except those with abnormal echocardiographic findings. Outcome was based on the presence or absence of coronary artery involvement.

### Statistical analysis

Data was analyzed by using Excel Sheet 2010. Quantitative variables such as age and laboratory values were expressed as means with range, and qualitative variables such as gender, clinical and echocardiographic findings were expressed as percentages. Fisher exact test was used for the comparative analysis of categorical variables and Mann-Whitney test was used for the comparison of continuous variables.

## RESULTS

Twenty-five children who met the clinical criteria for the diagnosis of KD, supported by laboratory or echocardiographic, were included in this study. Most children were males, below 60 months of age and slightly more presented in winter months (October-March) (Table-I). Complete KD was more common than incomplete KD (Table-I).

**Table-I:** Demographics, clinical and laboratory features of children with KD.

Characteristics	N (%), Mean, Range
***Demographics***	
Total children	25 (100)
Males	19 (76)
Mean age in months	43, 4-150
Age < 5 years	21 (84)
Presented in winter months	13 (52)
***Clinical features***	
Fever >5 days	20 (80)
Maculopapular rash	20 (80)
Oral mucosal changes	20 (80)
Conjuctivitis	20 (80)
Cervical lymphadenopathy	13 (52)
Extremity changes (edema, peeling)	10 (40)
***Laboratory features***	
White blood cell count (x 10^3^/µL)	18.6, 7.3-31.8
Platelet count (x 10^9^/µL)	585, 100-1030
Hemoglobin (gm/dl)	10.24, 7.6-12.8
ESR (mm/h)	59.5, 13-145
CRP (mg/L)	118.7, 7-281

The most prominent clinical features were fever, maculopapular rash, oral mucosal changes and conjunctivitis (Table-I). The number of febrile days ranged from 3-21 days at the time of diagnosis with mean temperature of 102.5°C (100-105°C). The mean interval between onset of fever and diagnosis of Kawasaki disease was 9 days. Laboratory investigations showed a high white blood cell count, platelet count, ESR and CRP (Table-I).Most children received IVIG within 10 days of fever (Table-II). Defervescence was achieved within 48 hours in all children. None of the children required a second dose of IVIG.

**Table-II:** Echocardiographic findings and outcome of children with KD.

Outcomes	N (%, Range)
Defervescence within 48 hours	25 (100)
IVIG within 10 days	15 (60)
***Initial echocardiography at diagnosis***	
Normal	17(68)
Abnormal	8 (32)
***Follow up echocardiography (2weeks)***	
Normal	17 (68)
Abnormal	8 (32)
***Abnormal echocardiographic features***	
Complete KD*	2 (25)
Incomplete KD**	6 (75)
***Patients with abnormal echocardiography showing CAA†***	
Coronary artery dilatation	5 (62.5)
Coronary artery aneurysm	1 (12.5)
***Characteristics of patients with abnormal echocardiography***	
Mean age in months	36 (4-120)
Males	6 (75)
Number of days of illness	7.8 (3-13)

† CAA coronary artery abnormalities,

* Patients with complete KD who showed abnormal echocardiographic findings,

** Patients with incomplete KD who showed abnormal echocardiographic findings.

Eight children (32%) had initial abnormal echocardiographic findings (Table-II). They were started on high dose aspirin. Out of these, the two patients with complete KD received a high dose of aspirin for 14 days, while the other patients received high dose of aspirin for three weeks. This difference in treatment was based on the higher risk of patients with incomplete KD developing coronary artery abnormalities. After that, it was continued in antiplatelet dose for 6–8 weeks, except the patient with persistent coronary artery aneurysm who continued to receive dual antiplatelet therapy with aspirin and clopidogrel.

The patient with complete KD who developed coronary complications had echocardiographic findings of mild left coronary artery (LCA) dilatation of 3 mm, normal right coronary artery (RCA) and circumflex artery (CA) initially. On follow up echo after 2 weeks it showed three fusiform dilatations of the LCA of 3 mm each. This child remained on low dose aspirin for 8 weeks till resolution of echocardiographic findings.

One more child with complete KD had a history of aortic stenosis. He developed flow turbulence across the aortic valve with peak gradient of 30-40 mmHg and mild aortic regurgitation but no coronary artery abnormality.

One child with incomplete KD developed significant fusiform aneurysm of the left main coronary artery (LMCA) and left anterior descending (LAD) artery 6 mm and 3.48 mm respectively and saccular aneurysm of proximal right coronary artery (RCA) 5.5 mm. On follow up, the coronary diameters improved to LMCA of 3.2 mm, LAD 3.2 mm and RCA 2.8 mm. This patient is still on follow up with clopidogrel and aspirin continued. Three other children with incomplete KD developed coronary artery dilatations only, which resolved by six months.

Another child with incomplete KD presented in a state of cardiogenic shock. He was intubated and remained on ionotropic support in the intensive care unit for two days. His initial echocardiogram showed an ejection fraction (EF) of 41% with left ventricular dysfunction and normal coronary arteries. This child’s follow up echo after 3 weeks was normal.

Furthermore, one child with incomplete KD showed a small restrictive ventricular septal defect on initial echo. His follow up echo after two weeks showed LMCA 3.04 mm, normal LAD, CA, RCA. This patient was lost to follow up after 8 weeks.

Most of the patients with CAA were male. Mean age was 36 months although extremes of ages (4-150 months) were seen. Duration of illness at presentation was above five days (Table-II). Eighteen children (72%) came for follow up for > 6 months. Except for one, all children eventually had normalization of echocardiographic findings (96%).

The features of children with complete KD versus incomplete KD were compared (Table-III). There was statistically significant difference in terms of age of presentation, white blood cell count, platelet count and echocardiographic findings.

**Table-III:** Comparison of children with complete versus incomplete KD.

Characteristics	Complete KD (%)	Incomplete KD (%)	P-value
Number of patients	18 (72)	7 (28)	
Number of male patients	12 (63)	7 (37)	0.10
Duration of fever (days)	8.8	10	0.06
Mean age (months)	57.2	22.7	0.04
Mean WBC count (x 10^3^/µL) †	13.2	21.8	0.01
Mean platelet count (x 10^9^/µL)	404.7	477.5	0.05
Mean CRP (mg/L) ††	98.9	127.3	0.10
Number of patients with abnormal echo findings	2 (25)	6 (75)	0.001

† WBC white blood cell count, ††C-reactive protein.

## DISCUSSION

The exact incidence of KD in Pakistan is not known. Singh et al showed the incidence of KD in India is 4.5 per 100 000 in children <15 years of age^7^ compared to Japan where the incidence is 265 per 100 000 children <5 years of age.^5^ KD is known to occur in children in Pakistan, as described by Alam et al in 2012.^8^ Mostly the features are as described in the literature. KD was seen to be more common in boys (76%) in our study which was consistent with other studies.^1^,^10^,^11^ Worldwide, KD occurs in two peak seasons of summers and winters.^12^ In this study, a slightly higher number of the patients (52%) were diagnosed during the winter months, which has been shown in other studies.^13^ However, peak incidence was found in other seasons too.^7^,^10^,^11^,^13^,^14^

Fever was the most consistent finding in this study along with maculopapular rash, oral mucosa changes and conjunctivitis, followed by cervical lymphadenopathy, while changes in the extremities such as edema and peeling was the least significant sign. These were consistent with the other studies.^10^,^11^,^14^ Routine laboratory values (white blood cell count, platelet count, ESR and CRP) were also similar to other studies.^7^,^10^,^11^,^14^

The frequency of cardiac involvement in KD is different in different countries such as Thailand (6.2%)^15^ Oman (25%)^16^ and Turkey (33%).^17^ Sonobe et al. noted the prevalence of coronary artery abnormality in complete KD was 14.2%, and 18.4% in incomplete KD.^18^ In this study, coronary artery involvement was 32% but the occurrence in incomplete disease was high (75%). Only one study addressing cardiovascular involvement in KD has been published from Pakistan in which coronary artery involvement was found to be 41% of the population included.^8^ It concluded that presentation after 10 days of illness, male gender, age <1 year or >5 years, and resistance to initial IVIG treatment have a higher risk of coronary artery involvement.^8^,^19^ Although, the sample size in this study was too limited to evaluate these factors in detail, similar results were seen. In this study, children had a good response to IVIG and aspirin, with resolution of signs, symptoms and abnormal laboratory parameters within 48 hours. There was no need of a second dose of IVIG in any patient. Cardiac abnormalities resolved in all children except one who needed long-term antiplatelet therapy. Overall, prognosis of the disease was good and morbidity was low.

In comparing the features of complete KD versus incomplete KD, our findings showed that children with incomplete KD were all males, of a younger age group, had a higher white cell and platelet count, and more likely to develop coronary artery abnormalities. These findings are similar to some other studies.^18^,^20^

### Limitations of the study

As this is a retrospective study it has its limitations. Children with missing data had to be excluded. One third of patients was lost to follow up. Since we are not a public hospital, we do not get the bulk of patients from the community, therefore, our study is not a representative of the burden of Kawasaki disease in our area or nationwide. Also, IVIG is an expensive treatment. Children who presented to our hospital could afford the treatment, either out-of-pocket or through insurance. This may be a reason why we saw fewer coronary artery involvements. A large multicenter study is necessary to investigate the overall incidence, potential risk factors, magnitude and prognosis of KD disease in our population.

## CONCLUSION

Complete KD was present in 72% of our cohort of children. Coronary artery abnormalities were present in one third of these children, at younger age and more common in those with incomplete KD but recovered in most. KD must be considered in the differential diagnosis of any child presenting with prolonged and unexplainable fever.

### Authors’ Contribution:

**SS:** Data collection, manuscript writing, statistical analysis.

**SSO:** Concept and design, editing, statistical analysis.

**EAK:** Concept and design, editing, final review, accountable for accuracy
